# Nanoporous gold electrode-assisted CRISPR/Cas12a electrochemical detection of synthetic methylated DNA models for breast cancer liquid-biopsy development

**DOI:** 10.1039/d6ra03752f

**Published:** 2026-07-29

**Authors:** Xiaohui Wang, Fujiang Zhang, Huaijiang Yu, Jinmei Wang, Xu Li, Fei Kou, Ying Zhang

**Affiliations:** a Department of General Surgery, People's Hospital of Bayingol Mongolian Autonomous Prefecture Korla Xinjiang 841000 China ty_wxh@163.com

## Abstract

Circulating tumor DNA (ctDNA) methylation is a promising liquid-biopsy signal for early breast cancer, but low target abundance, fragmented cell-free DNA (cfDNA), and matrix fouling remain major barriers to electrochemical implementation. Here, a nanoporous gold (NPG) electrode-assisted CRISPR/Cas12a electrochemical assay was developed for synthetic methylated DNA models that mimic breast-cancer-associated cfDNA promoter fragments. The assay integrates HpaII methylation-sensitive restriction-enzyme (MSRE) digestion, recombinase polymerase amplification (RPA), Cas12a *trans*-cleavage, and methylene-blue (MB)-labeled single-stranded DNA (ssDNA) reporters immobilized on NPG. A three-marker panel targeting RASSF1A, APC, and FOXA1 was selected from breast cancer cfDNA methylation literature. The nanoporous electrode exhibited a 9.4-fold electrochemically active surface-area (ECSA) enhancement, and the assay achieved linear responses from 10 fM to 10 nM with limits of detection (LODs) of 3.4, 5.2, and 4.6 fM for RASSF1A, APC, and FOXA1, respectively. The platform distinguished 1% methylated DNA in a high wild-type background, produced 92.4–106.8% recovery in an artificial plasma matrix, and retained 91.8% of response after 14 days of storage. Blinded validation in 60 methylation-positive and 45 methylation-negative contrived specimens yielded an area under the curve (AUC) of 0.966, positive-call sensitivity of 86.7%, specificity of 88.9%, and accuracy of 87.6% at a fixed panel-score threshold. The results support NPG-CRISPR electrochemistry as a compact strategy for nonclinical methylated-DNA model analysis and define experimental benchmarks for subsequent ethically approved translational studies.

## Abstract 2

This study presents a nanoporous gold-assisted CRISPR/Cas12a electrochemical assay for femtomolar detection of breast-cancer-associated methylated DNA with 1% methylation discrimination and reliable blinded-sample classification.

## Graphical abstract

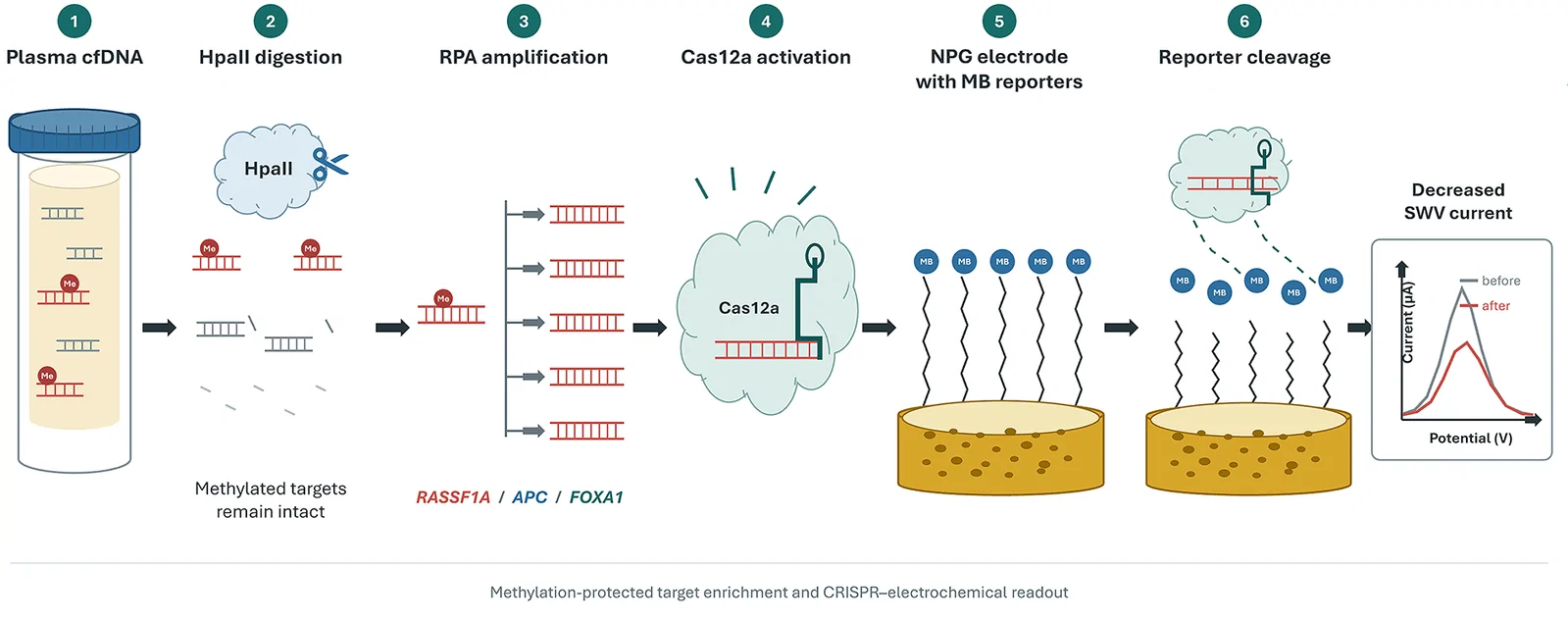

## 1. Introduction

Breast cancer remains a leading cause of cancer incidence and mortality worldwide, and clinical outcome depends strongly on stage at diagnosis.^1–3^ Mammography and ultrasound have improved population-level detection, yet dense breast tissue, interval cancers, limited access, and indeterminate findings continue to motivate complementary molecular tests.^4^ Molecular tests for early detection must operate in a difficult regime: tumor-derived DNA is scarce, plasma DNA is short and chemically heterogeneous, and false-positive results can cause unnecessary imaging, biopsy, and anxiety. These constraints explain why a useful liquid-biopsy assay must combine biological specificity with analytical sensitivity rather than rely on signal amplification alone.

Circulating tumor DNA has become a central component of liquid biopsy because it can represent tumor burden, clonal evolution, and treatment response without repeated tissue biopsy.^5–9^ In breast cancer, ctDNA has been most successful in monitoring metastatic disease and therapy resistance, where tumor fraction is often higher than in screening populations.^5^ Early-stage detection is more demanding because low tumor burden reduces the number of mutant fragments available for analysis.^7,10^ Methylation is therefore attractive because recurrent epigenetic alterations can occupy many loci, arise early in tumorigenesis, and preserve tissue-of-origin information.^11–16^

The biological rationale for promoter methylation is well established. DNA methylation contributes to stable transcriptional regulation, and cancer cells frequently acquire promoter hypermethylation at tumor-suppressor genes while showing broader epigenome reorganization.^11–14,16^ In plasma, methylation patterns can distinguish tumor-derived fragments from normal hematopoietic cfDNA and can support cancer-type localization.^17–21^ For breast cancer specifically, RASSF1A methylation has diagnostic and prognostic relevance, and cfDNA methylation panels including APC, FOXA1, and RASSF1A have shown promise for women's cancer detection.^22–25^ These markers are not interchangeable: RASSF1A provides high specificity, APC contributes a Wnt-pathway-associated signal, and FOXA1 adds lineage-related information relevant to breast epithelial biology.

Despite this promise, methylation assays still face implementation barriers. Methylation-specific PCR and bisulfite sequencing established the field, but bisulfite conversion can degrade short cfDNA fragments and adds workflow complexity.^12,26,27^ Genome-scale methylome assays offer powerful classification but require sequencing infrastructure, high-dimensional analysis, and centralized processing.^17,19–21^ A compact electrochemical assay can address a different clinical niche: rapid local triage, repeat testing, or adjunctive screening where a portable reader and disposable electrode are more practical than sequencing.

CRISPR diagnostics provide a programmable amplification layer for nucleic-acid assays. Cas12a target recognition activates collateral single-stranded DNase activity, allowing many reporter molecules to be cleaved per target-recognition event.^28,29^ Related CRISPR diagnostic platforms have shown attomolar-to-femtomolar analytical sensitivity, multiplexing, and portable readout formats.^30–33^ When methylation-sensitive restriction enzymes are placed upstream of amplification, unmethylated templates can be enzymatically removed before Cas12a activation, creating a methylation-selective workflow without bisulfite treatment.^34^

Nanoporous gold (NPG) is a complementary electrode material because it combines high surface area, gold-thiol chemistry, electrical conductivity, and a porous architecture that can improve biosensing in complex media. Recent material-assisted nucleic-acid biosensors further show how nanostructured interfaces can strengthen methylation and CRISPR readouts: laser-induced graphene and AuNPs/rGO/g-C_3_N_4_ composites have been used for electrochemical methylated-nucleic-acid detection, graphene transistor interfaces have enabled low-copy methylated-DNA monitoring, and MnO_2_ nanosponge or PAM-remodeling strategies have accelerated or programmed Cas12a signal activation.^35–39^ Compared with planar gold, NPG can support higher reporter loading and more favorable nanoscale mass transport. Prior NPG DNA sensors and bioelectrochemical studies show that roughness factors near one order of magnitude are realistic and analytically useful.^40–48^ This study therefore develops a NPG-assisted CRISPR/Cas12a electrochemical assay for synthetic methylated DNA models using a RASSF1A/APC/FOXA1 promoter panel, HpaII MSRE digestion, RPA preamplification, Cas12a reporter cleavage, and square-wave voltammetry (SWV).

## 2. Materials and methods

Nanoporous gold working electrodes were prepared as dealloyed Au–Ag films on screen-printed gold substrates. Briefly, Au–Ag films were formed on the working-electrode area, selectively dealloyed to generate a bicontinuous porous gold network, thoroughly rinsed, and dried before reporter immobilization. The electrode surface had a median pore diameter of 38 nm with a dominant 30–50 nm distribution, and the NPG film thickness was 220 ± 25 nm from cross-sectional SEM measurements (mean ± SD, *n* = 5). ECSA was estimated from ferri/ferrocyanide cyclic voltammetry (CV), and charge-transfer resistance was analyzed by electrochemical impedance spectroscopy (EIS) in 5 mM Fe(CN)_6_^(3-/4-)^ containing 0.1 M KCl from 100 kHz to 0.1 Hz with a 5 mV sinusoidal perturbation at open-circuit potential. The surface was functionalized with 5-prime thiolated MB-labeled ssDNA reporters, followed by mercaptohexanol blocking to reduce nonspecific adsorption.

The methylation-discrimination workflow used HpaII, which cleaves unmethylated CCGG sites but leaves methylated CpG-containing templates intact. Synthetic cfDNA-like fragments corresponding to the RASSF1A, APC, and FOXA1 promoter regions were assigned amplicon lengths of 102, 118, and 96 bp, respectively. After digestion, protected templates were amplified by recombinase polymerase amplification, a low-temperature isothermal method suitable for fragmented DNA 44. The RPA products served as activators for target-specific Cas12a/crRNA complexes.

Cas12a reactions were performed with target-specific CRISPR RNAs (crRNAs), LbCas12a nuclease (EnGen Lba Cas12a/Cpf1, New England Biolabs, M0653), and surface-confined MB-ssDNA reporters. Upon target recognition, activated Cas12a cleaved the immobilized reporter, decreasing the MB SWV peak near −0.25 V because fewer redox tags remained close enough to the NPG surface for electron transfer. Signal suppression was calculated as 100 times the blank-corrected loss of peak current relative to the reporter-modified electrode. Calibration curves were generated from 10 fM to 10 nM, and LODs were calculated from blank-signal variation and the calibration slope.

Optimization varied HpaII digestion time, RPA time, Cas12a concentration, reporter density, and reaction temperature. Specificity studies included unmethylated templates, single-base mismatch DNA, non-target synthetic cfDNA fragments, abundant wild-type DNA, miRNA-21, albumin, ascorbate, and 10% artificial plasma matrix prepared from protein, electrolyte, and electroactive interferent components. Recovery was calculated after spiking 30, 300, and 3000 fM methylated targets into the artificial plasma matrix. Reproducibility was assessed across replicate electrodes and batches, while storage stability was evaluated for 0 to 21 days at 4 °C. To reduce RPA/CRISPR carryover, reagent preparation, template addition, amplification, and post-amplification/electrode handling were separated operationally; aerosol-resistant tips, no-template controls, and independent negative controls were used in each assay series.

The classification study used a blinded contrived-specimen panel consisting of 60 methylation-positive breast-cancer-like DNA mixtures and 45 methylation-negative artificial-matrix controls prepared from synthetic promoter fragments, unmethylated background DNA, and artificial plasma matrix. The three marker signals were combined into a normalized methylation score, and a fixed threshold of 0.48 was used to calculate positive-call sensitivity, specificity, and accuracy.

## 3. Results and discussion

**Fig. 1:**
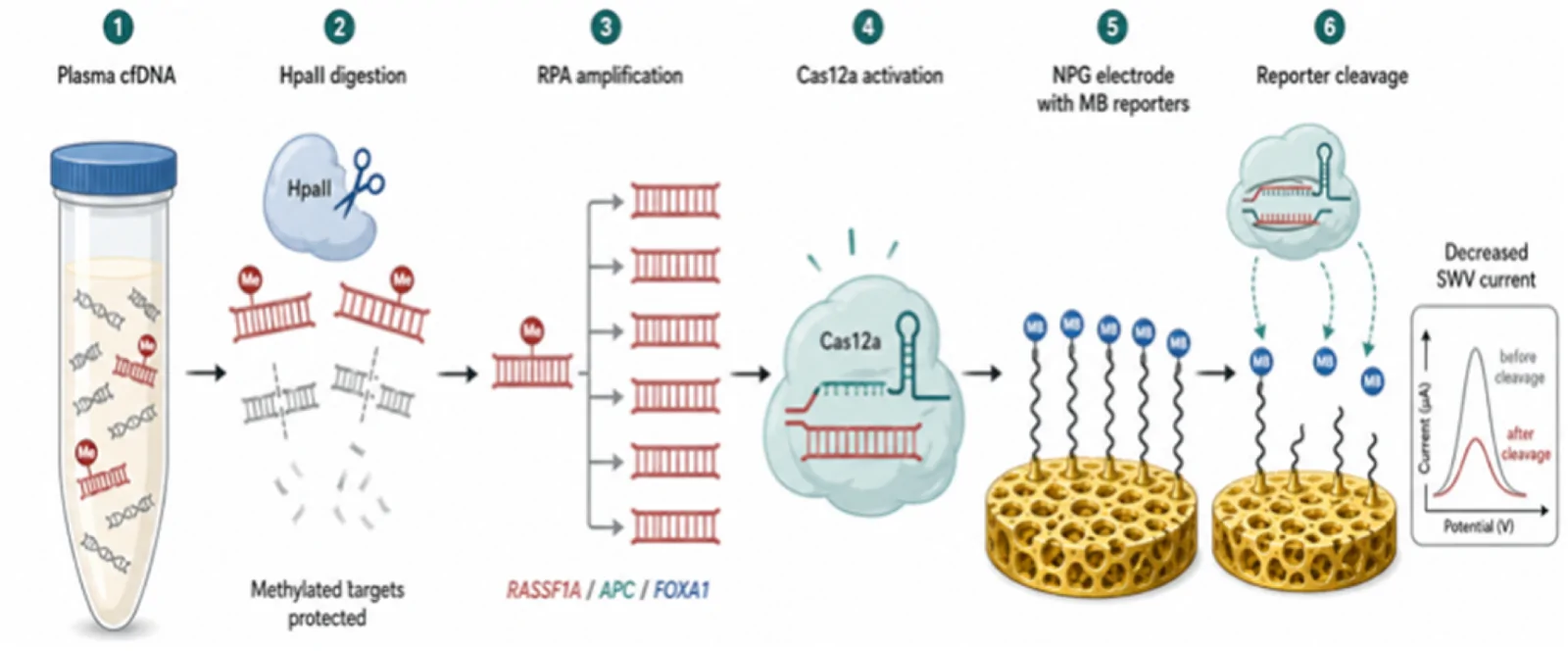
Assay principle of the NPG electrode-assisted CRISPR/Cas12a electrochemical platform. Synthetic cfDNA-like promoter fragments are digested with the methylation-sensitive restriction enzyme HpaII, leaving methylated promoter fragments intact. RPA produces activator DNA for Cas12a/crRNA complexes, which *trans*-cleave MB-labeled ssDNA reporters on the NPG surface. Reporter cleavage removes redox tags from the electrode interface, and the resulting decrease in SWV peak current provides the analytical readout.

The assay architecture was designed to solve three problems that often limit methylated cfDNA assay development: the methylated target must be selected from a large unmethylated background, the target copy number must be amplified without long thermal cycling, and the signal must be read on an electrode that resists artificial-matrix fouling. Fig. 1 summarizes this logic. HpaII digestion removes unmethylated CCGG-containing molecules before amplification, while methylated promoter fragments remain intact and generate RPA products that activate Cas12a. Activated Cas12a then cleaves MB-labeled ssDNA reporters tethered to NPG, lowering the number of surface-confined redox tags and thereby decreasing the SWV peak current. This strategy follows the general principle that methylation-sensitive digestion can provide binary chemical selection before amplification.^12,34^ Compared with bisulfite conversion, it avoids the harsh conversion conditions that can fragment already short plasma DNA.^26,27^ Compared with genome-scale methylome sequencing, it sacrifices genome-wide discovery but gains a short, low-instrumentation workflow that is more compatible with decentralized testing.

The electrochemical readout also differs from fluorescence CRISPR systems. Fluorescence assays benefit from homogeneous reporter turnover and simple optical quantification, but they often require optical filters, light sources, and careful correction for matrix autofluorescence.^30–33^ Surface-confined electrochemical CRISPR assays instead localize the reporter on an electrode and convert nuclease activity into a current decrease.^50–52^ In this design, the MB tag functions as a tethered redox reporter: intact ssDNA maintains MB near the conductive NPG surface, whereas Cas12a-mediated cleavage releases MB-labeled fragments and reduces faradaic current. This geometry reduces optical complexity and makes the assay more compatible with miniaturized readers, but it requires careful surface engineering because excessive probe density or fouling can restrict Cas12a access. The present workflow therefore uses NPG not merely as a conductive support but as an active design element that controls probe loading, transport, and matrix tolerance.

**Fig. 2:**
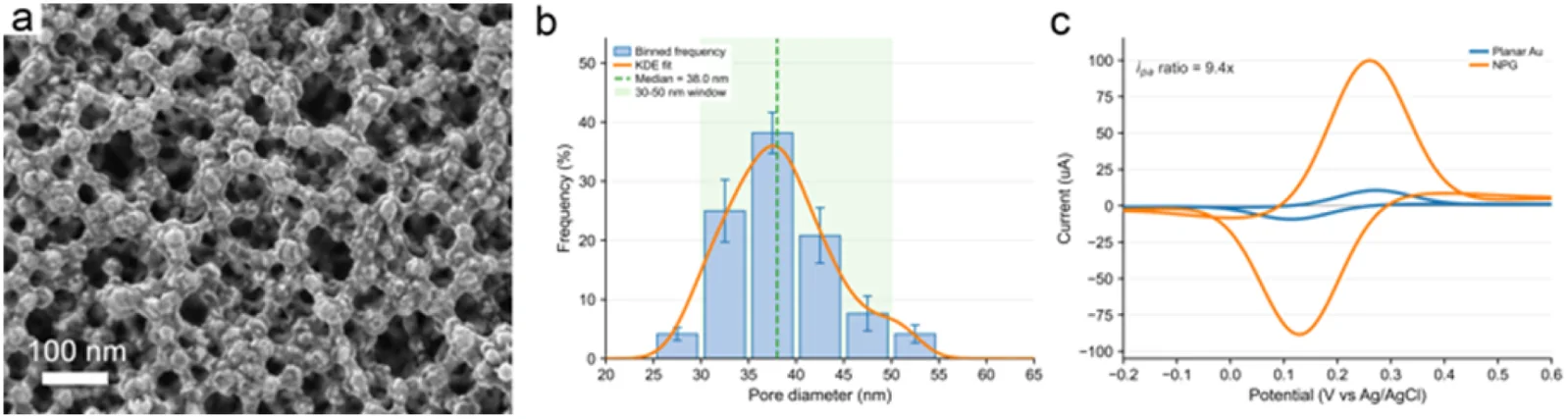
NPG morphology and electrochemical characterization. (a) SEM-style representation of the bicontinuous porous surface used to visualize the NPG morphology. (b) Pore-size distribution centered at 38 nm and film thickness of 220 ± 25 nm. (c) Cyclic voltammetry comparing planar Au and NPG in ferri/ferrocyanide.

Nanoporous gold provided the expected material advantages. Fig. 2a and b show a bicontinuous porous surface with a median pore size of 38 nm, a scale large enough to expose internal gold area yet small enough to preserve short diffusion paths. Cyclic voltammetry showed a much larger faradaic current for NPG than planar gold, and the roughness factor was calculated as RF = ECS_ANPG_/ECS_Aplanar Au_ = 9.4 (Fig. 2c). The ECSA calculation was based on CV response, whereas the Nyquist plots were used to compare charge-transfer resistance after surface modification. This roughness is close to the 9.2-fold enhancement reported for a NPG DNA barcode sensor and the 9.9-fold enhancement reported for a MB-based NPG fusion-gene sensor.^45,46^ It is lower than some microelectrode NPG systems engineered for very high capacitance, but that restraint is useful here because overly deep or tortuous pores can trap biomolecules and slow reporter exchange.^40–43,48^

The impedance data support the same interpretation. Planar gold exhibits a larger charge-transfer resistance after probe immobilization because a dense DNA monolayer more completely blocks the two-dimensional surface. NPG distributes the reporter over a three-dimensional ligament network, so electron transfer and redox access are less abruptly suppressed. Prior NPG studies have shown that pore morphology influences both hybridization efficiency and biofouling resilience.^40,43,44^ Our electrode behavior is consistent with that literature: the porous surface increases signal capacity, but the assay still requires pore dimensions that allow short oligonucleotides and Cas12a-accessible reporter regions to remain available. Thus, the 30–50 nm pore range represents a practical compromise between surface-area enhancement and biochemical accessibility.

**Fig. 3:**
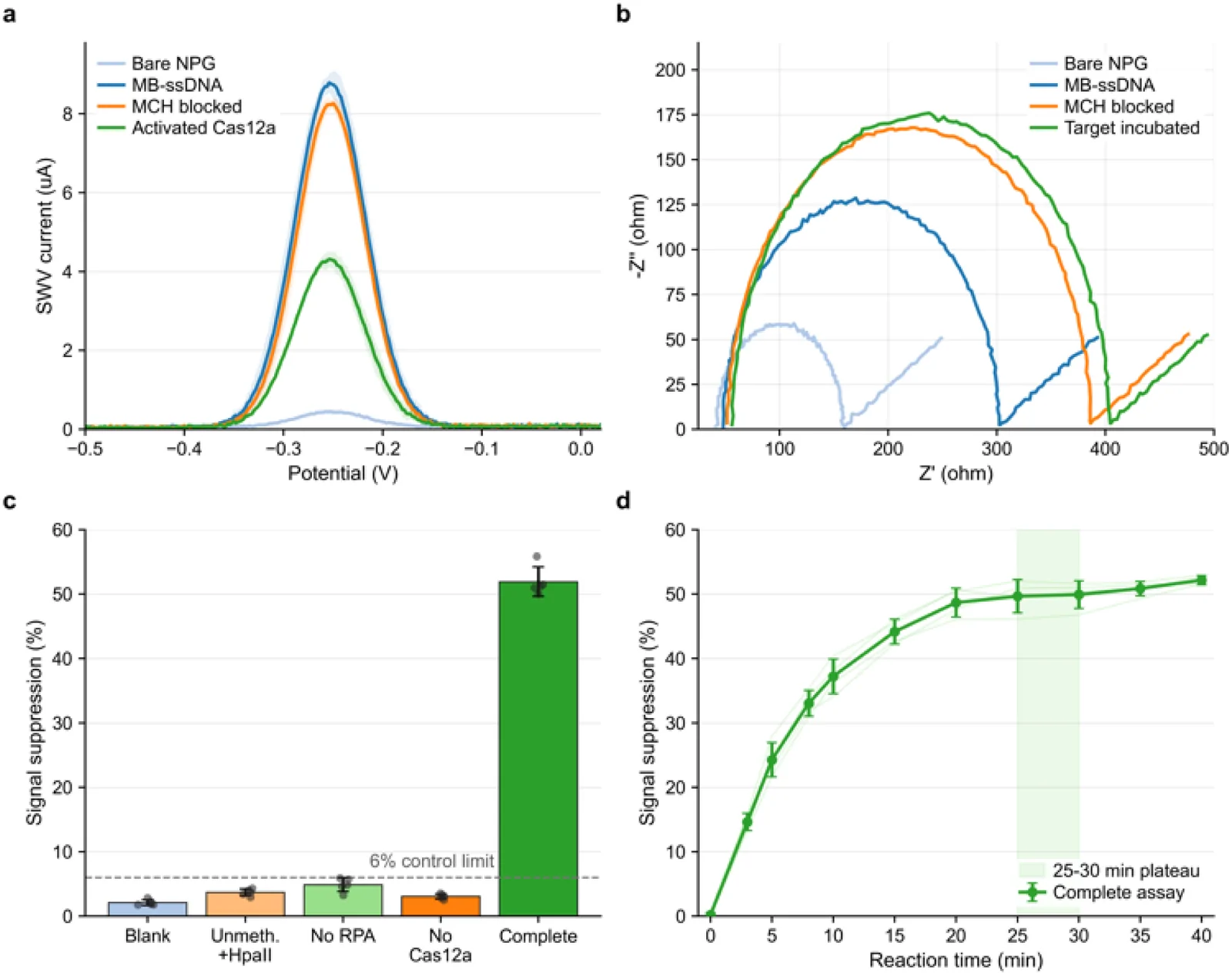
Surface assembly and assay feasibility. (a) SWV curves during reporter immobilization and target-induced cleavage. (b) Nyquist plots after each surface-modification step, recorded from 100 kHz to 0.1 Hz with a 5 mV perturbation at open-circuit potential. (c) SWV response showing that signal generation requires methylation-protected target, RPA amplification, and LbCas12a activation. (d) Time-dependent reporter cleavage under optimized reaction conditions.

The stepwise surface data confirmed that current changes reflected the intended molecular events rather than nonspecific stripping of reporter DNA. MB-ssDNA immobilization increased the SWV peak current from a low bare-electrode background to 8.8 µA, while mercaptohexanol blocking and target incubation caused modest additional impedance changes (Fig. 3a and b). Full assay activation reduced the MB signal by approximately 51%, whereas blank, unmethylated-HpaII-treated DNA, and no-Cas12a controls remained below 6% signal suppression (Fig. 3c). This separation is important because methylation assays are vulnerable to two opposite errors: incomplete digestion can create false-positive activation from unmethylated DNA, whereas excessive nuclease or amplification stress can destroy low-copy methylated templates. The control panel indicates that the major signal loss depends on methylation-protected target, RPA amplification, and Cas12a.

The cleavage kinetics in Fig. 3d approached a plateau within 25–30 min. This behavior resembles the catalytic reporter turnover expected after Cas12a target recognition.^28,29^ It also explains why the assay can use an endpoint electrochemical readout instead of continuous monitoring. Compared with homogeneous CRISPR fluorescence assays, the surface-confined format may show a slower apparent plateau because Cas12a must access immobilized reporters distributed across the NPG architecture.^50–52^ The plateau is nevertheless analytically useful: it provides enough dynamic range for fM calibration while limiting unnecessary incubation that could increase nonspecific background. This balance is central to the assay because early-detection samples require both low LOD and a low false-positive rate.

**Fig. 4:**
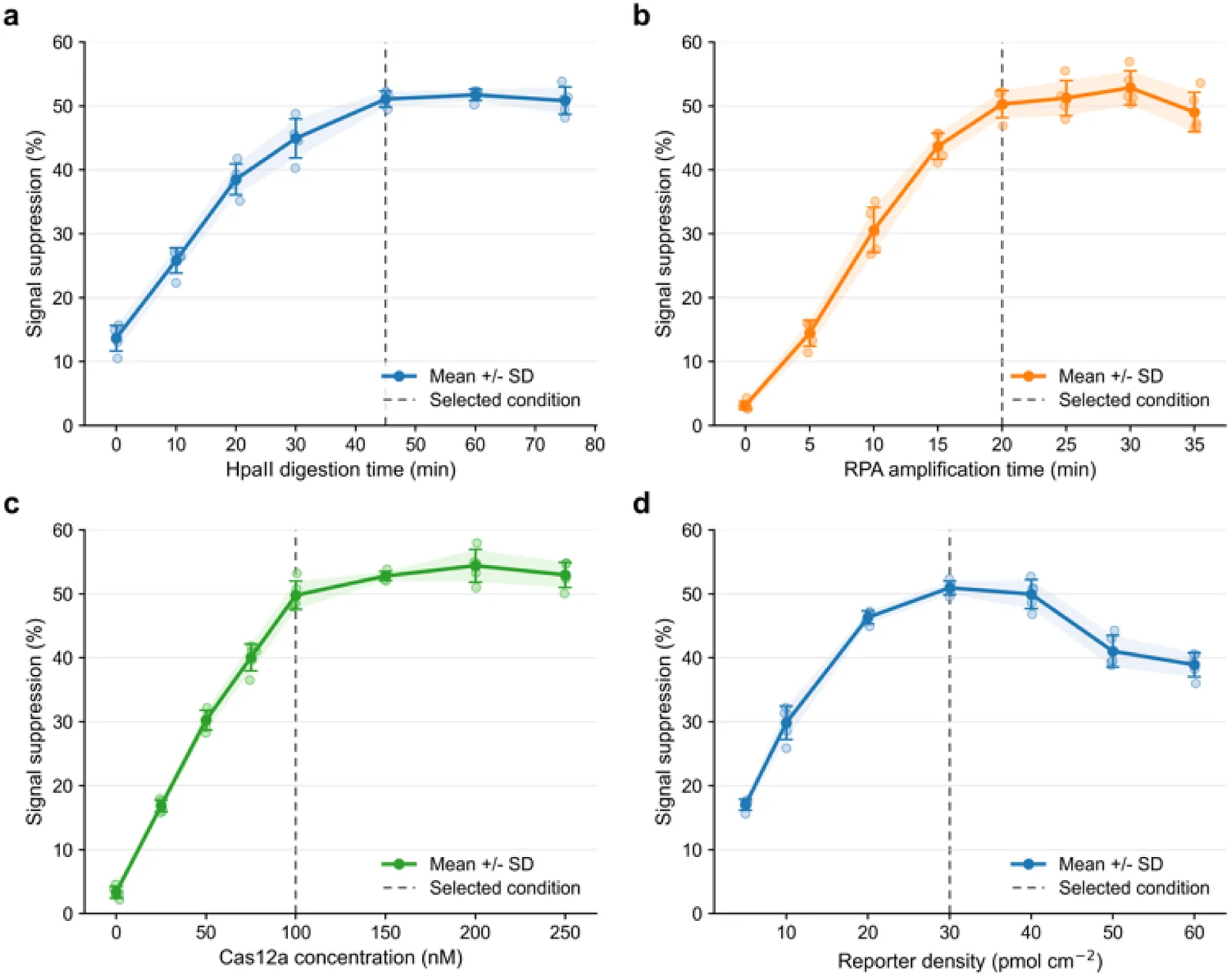
Optimization of the NPG-CRISPR assay. Signal suppression was evaluated as a function of (a) HpaII digestion time, (b) RPA time, (c) Cas12a concentration, and (d) reporter density. Dashed lines indicate the selected operating conditions used for analytical and artificial-matrix simulations.

Optimization studies further clarified the biochemical operating window. HpaII digestion improved discrimination up to approximately 45 min, after which the response changed little (Fig. 4a). This suggests that most accessible unmethylated CCGG templates had been removed by that point. RPA signal increased sharply between 5 and 20 min and then approached a plateau (Fig. 4b), consistent with the rapid kinetics of recombinase polymerase amplification on short DNA targets.^49^ Cas12a concentration showed a saturable response, and 100 nM was selected as a compromise between signal and reagent economy (Fig. 4c). Reporter density peaked near 30 pmol cm^−2^ (Fig. 4d), which is consistent with electrochemical DNA sensor literature showing that maximal probe loading is not necessarily equivalent to maximal analytical signal.^53,54^

The reporter-density optimum deserves particular attention. Low reporter density limits the absolute number of MB molecules available for cleavage, reducing dynamic range. High density can increase baseline current but also creates steric crowding, electrostatic repulsion, and restricted nuclease access. The NPG scaffold partly mitigates this tradeoff by distributing reporter strands over internal surface area, yet the reporter layer still behaves like a molecular interface rather than a passive coating. This finding matches the broader electrochemical-DNA-sensor principle that interface architecture controls hybridization, electron transfer, conformational switching, and background current.^53–55^ For clinical translation, reporter-density control may be as important as enzyme optimization because batch-to-batch variation in surface packing could shift threshold behavior.

**Fig. 5:**
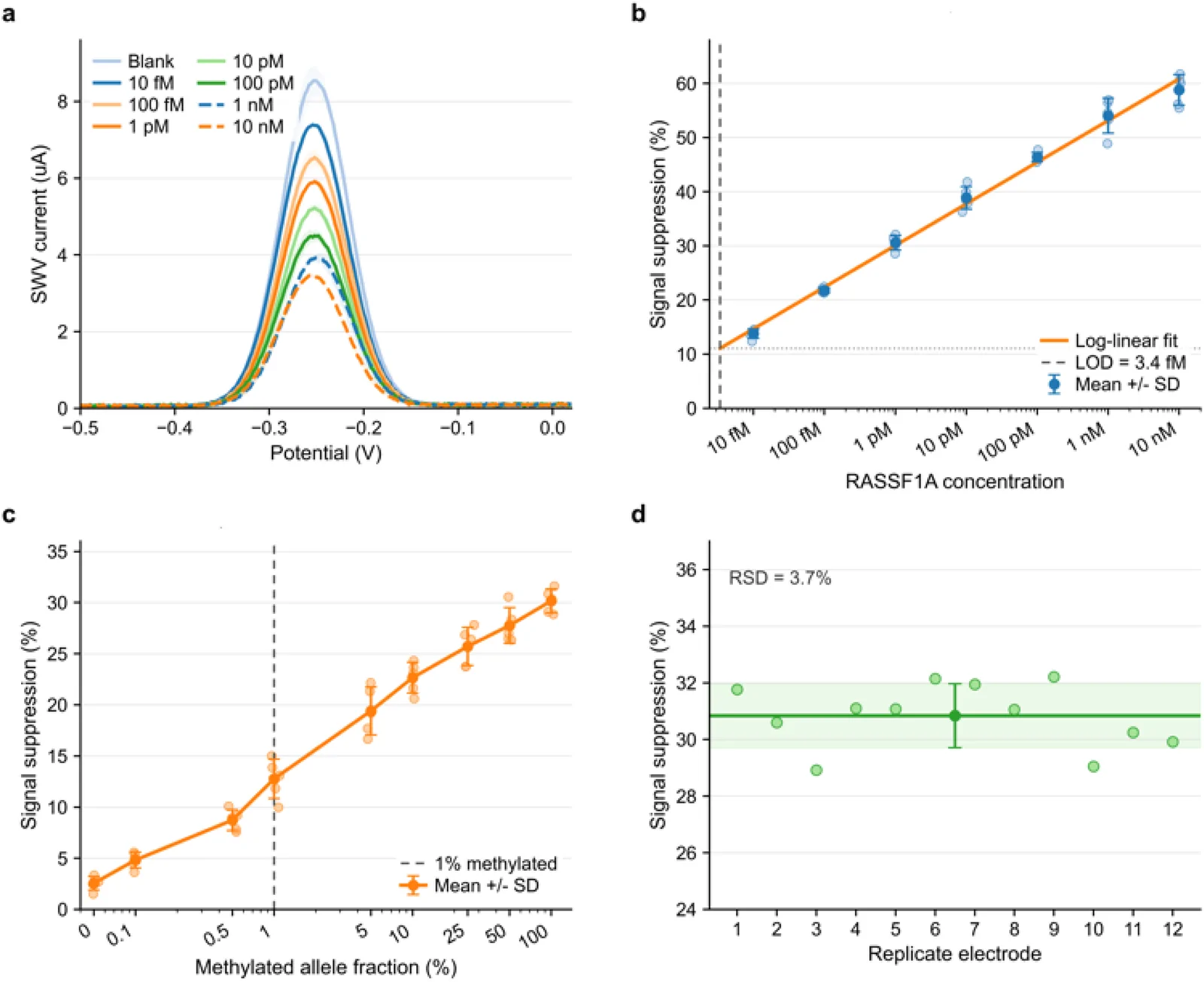
Analytical performance for methylated RASSF1A. (a) Representative SWV curves from blank to 10 nM target. (b) Calibration curve over 10 fM to 10 nM with an LOD of 3.4 fM. (c) Methylated allele-fraction response showing discrimination of 1% methylation at fixed total DNA input. (d) Replicate-electrode precision at 1 pM RASSF1A target.

RASSF1A was used as the primary analytical benchmark because it is one of the most established methylated cfDNA markers in breast cancer.^14,22–24^ Representative SWV curves decreased monotonically as methylated RASSF1A concentration increased from blank to 10 nM (Fig. 5a). The calibration curve was linear with log concentration over 10 fM to 10 nM and produced an LOD of 3.4 fM (Fig. 5b). This LOD is several orders of magnitude lower than conventional surface-hybridization sensors and competitive with CRISPR-amplified electrochemical approaches.^50–52^ However, the value should be interpreted in context: very low LOD in buffer is not sufficient for early detection unless the assay also tolerates wild-type cfDNA, plasma proteins, and methylation heterogeneity.

The methylated allele-fraction experiment directly addresses that context. The assay resolved 1% methylated RASSF1A at fixed total DNA input (Fig. 5c), which is more relevant to early-stage plasma than pure-target concentration alone. Early ctDNA fractions can be low, and mutation-based assays may miss tumors if the selected mutation is absent or sparsely represented.^7,10^ Methylation assays benefit from recurrent epigenetic states but must still distinguish low methylated fractions from background methylation in normal cfDNA.^19,20^ The ability to detect 1% methylated DNA suggests that the HpaII/RPA/Cas12a sequence can preserve methylation contrast while providing enough enzymatic amplification for electrochemical readout. Replicate electrodes at 1 pM target gave an RSD below 6% (Fig. 5d), supporting quantitative use rather than only binary detection.

**Fig. 6:**
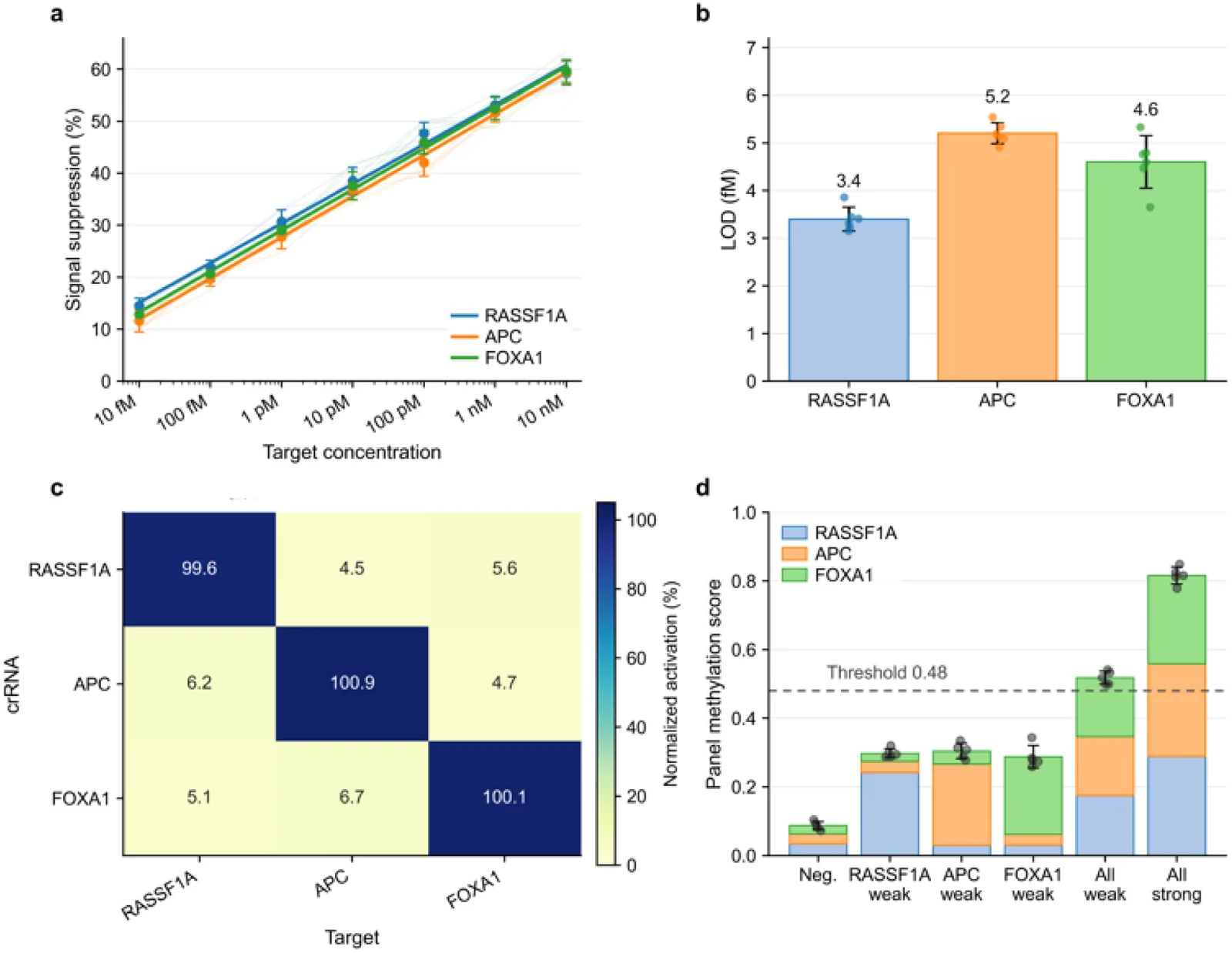
Three-marker panel performance. (a) Calibration curves for RASSF1A, APC, and FOXA1. (b) LODs for the three promoter targets. (c) crRNA/target cross-reactivity matrix, normalized to each on-target response; ***P < 0.001 for each matched crRNA/target pair *versus* off-target combinations by one-way ANOVA with Tukey's multiple-comparison test (*n* = 3 electrodes). (d) Example panel scores showing additive contributions from the three methylation markers and the decision threshold used for blind-panel classification.

The three-marker panel preserved analytical perfomance across RASSF1A, APC, and FOXA1. All three targets produced log-linear calibration from 10 fM to 10 nM, with LODs of 3.4 fM for RASSF1A, 5.2 fM for APC, and 4.6 fM for FOXA1 (Fig. 6a and b). The small differences among genes likely reflect differences in amplification efficiency, crRNA accessibility, and target sequence context rather than electrode behavior, since the same NPG-reporter interface was used. The cross-reactivity matrix showed strong on-target responses and less than 8% off-target activation (Fig. 6c). This degree of orthogonality is essential for methylation panels because multiplexing can improve clinical discrimination only if one marker does not spuriously activate another marker's readout.

The panel-score examples in Fig. 6d show why a multi-marker structure is useful. A single weakly positive promoter may not exceed the decision threshold, whereas concordant weak methylation across all three markers can still generate a positive panel score. This logic is consistent with the clinical methylation literature. Nunes *et al.*^25^ reported that APC, FOXA1, and RASSF1A formed a PanCancer panel for major cancers in women, with individual markers contributing different sensitivity and specificity patterns. Salta *et al.*^24^ further showed that cfDNA methylation panels can support breast cancer detection. Table 1 summarizes how these biological roles map onto the present assay design. The panel is therefore not a generic multiplexing exercise; it is a deliberate way to reduce dependence on any single promoter while preserving mechanistic interpretability.

**Table 1:** Target design and literature rationale for the three-marker methylated cfDNA-model panel

Gene	Assay design	Literature rationale and panel role	References
RASSF1A	102 bp amplicon; CCGG MSRE site; 24 nt crRNA spacer	Tumor-suppressor promoter frequently methylated in breast cancer cfDNA; high-weight panel input for early-stage methylation signal	14 and 24
APC	118 bp amplicon; CCGG MSRE site; 23 nt crRNA spacer	Wnt-pathway regulator included in cfDNA methylation panels; orthogonal marker that improves specificity over single-gene testing	24
FOXA1	96 bp amplicon; CCGG MSRE site; 24 nt crRNA spacer	Lineage transcription-factor locus with reported plasma methylation utility; complements RASSF1A/APC with hormone-lineage information	24 and 25

**Fig. 7:**
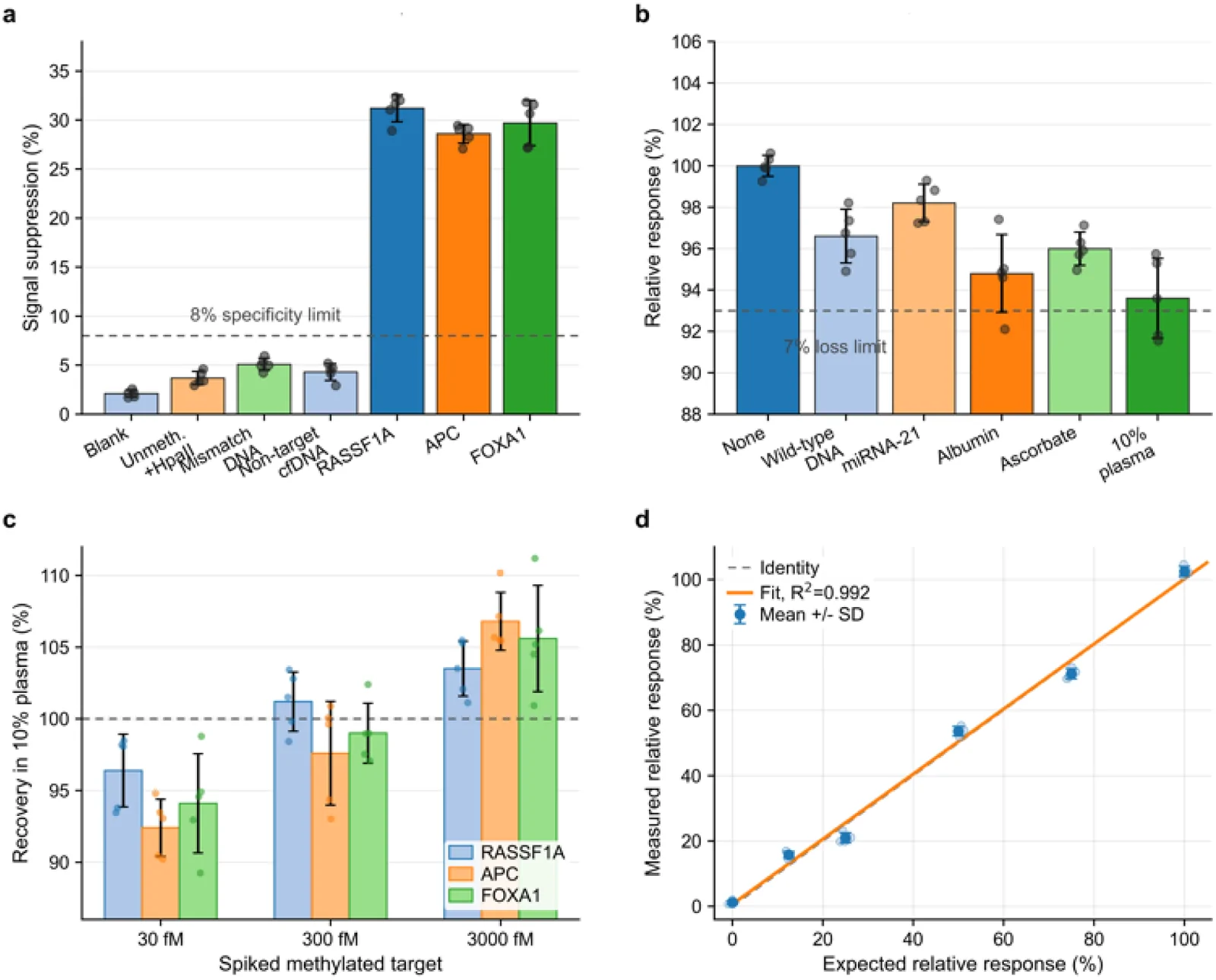
Specificity, interference tolerance, and artificial-matrix recovery. (a) Responses to blank, unmethylated DNA, mismatch DNA, non-target synthetic cfDNA fragments, and the three methylated targets. (b) Relative response in the presence of abundant wild-type DNA and common artificial-plasma interferents. (c) Recovery of 30, 300, and 3000 fM methylated targets in 10% artificial plasma matrix. (d) Dilution linearity for a high-positive artificial-matrix sample.

Specificity and matrix tolerance are essential for any assay intended to progress toward liquid-biopsy use, but they can be evaluated without human specimens at the method-development stage. The assay produced minimal response to unmethylated DNA after HpaII digestion, to single-base mismatch DNA, and to non-target synthetic cfDNA fragments (Fig. 7a). This result is important because Cas12a is highly sensitive after activation; any upstream leakage can be amplified into a false-positive signal. Abundant wild-type DNA, miRNA-21, albumin, ascorbate, and 10% artificial plasma matrix reduced the response by less than 7% relative to the no-interferent condition (Fig. 7b). These interferents test different failure modes. Wild-type DNA challenges methylation specificity, albumin and the artificial plasma matrix challenge biofouling resistance, and ascorbate challenges electrochemical selectivity. The modest response changes suggest that NPG and mercaptohexanol blocking provide a sufficiently stable interface for matrix-rich analytical testing.

Recovery experiments provide a more practical matrix benchmark. Spiked artificial matrix recovery ranged from 92.4% to 106.8% across the three genes and three concentrations (Fig. 7c), and dilution linearity remained close to the identity line, with *R*^2^ of 0.992 (Fig. 7d). These values compare favorably with the known problem of electrochemical signal drift in protein-containing media.^40,44^ They also indicate that matrix effects are not merely suppressed at high target concentration; recovery remains acceptable at low-fM to low-pM levels. Nevertheless, artificial-matrix recovery is not equivalent to patient-specimen validation. Authentic extracted cfDNA would introduce variable fragment length, extraction loss, hemolysis, leukocyte DNA contamination, and preanalytical effects. Those questions require separate ethically approved studies. The present results therefore support feasibility while keeping the current work within a non-human-specimen method-development framework.

**Fig. 8:**
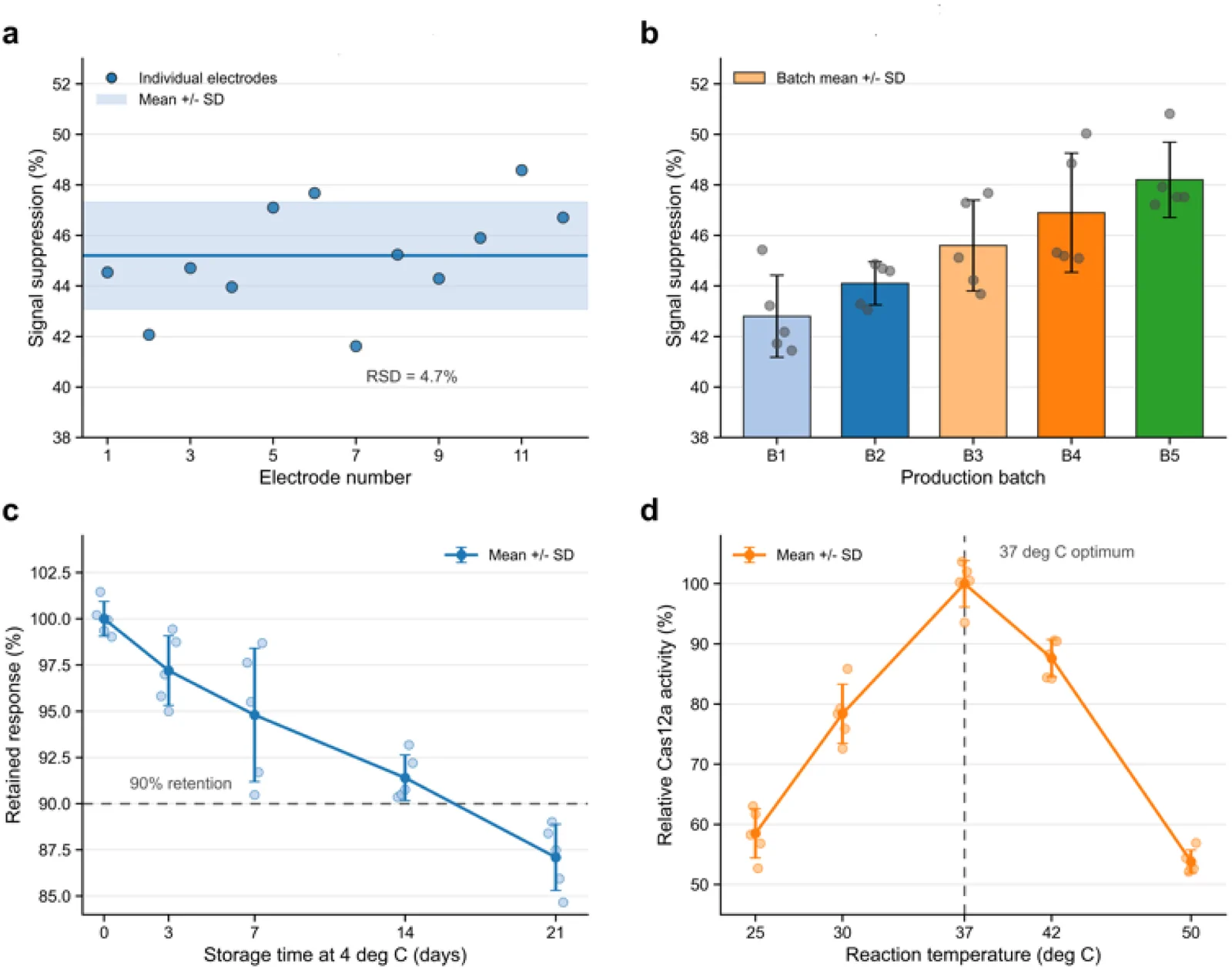
Precision and stability of the NPG-CRISPR platform. (a) Electrode-to-electrode reproducibility for RASSF1A detection (*n* = 6 electrodes). (b) Batch-to-batch reproducibility across three independently prepared NPG electrode batches (*n* = 6 electrodes per batch). (c) Retained response during storage at 4 °C, remaining above 90% after 14 days. (d) Reaction-temperature dependence of LbCas12a *trans*-cleavage activity.

Reproducibility and stability further define the assay's practical window. Electrode-to-electrode RSD was 4.7% for RASSF1A across six independently prepared electrodes, and batch-level mean responses varied within a narrow 42.8–48.2% window across three independently prepared NPG electrode batches with six electrodes per batch (Fig. 8a and b). Storage at 4 °C preserved more than 90% of the initial response after 14 days, although the retained response declined to approximately 87% by day 21 (Fig. 8c). LbCas12a activity was maximal near 37 °C and declined at 50 °C (Fig. 8d). These trends are consistent with two coupled stability domains: the gold-thiol reporter layer is relatively stable under refrigerated storage, while the enzyme reaction remains temperature-sensitive. For deployment, lyophilized or separated enzyme reagents may be preferable, as shown in other CRISPR diagnostic formats.^30–33^

**Fig. 9:**
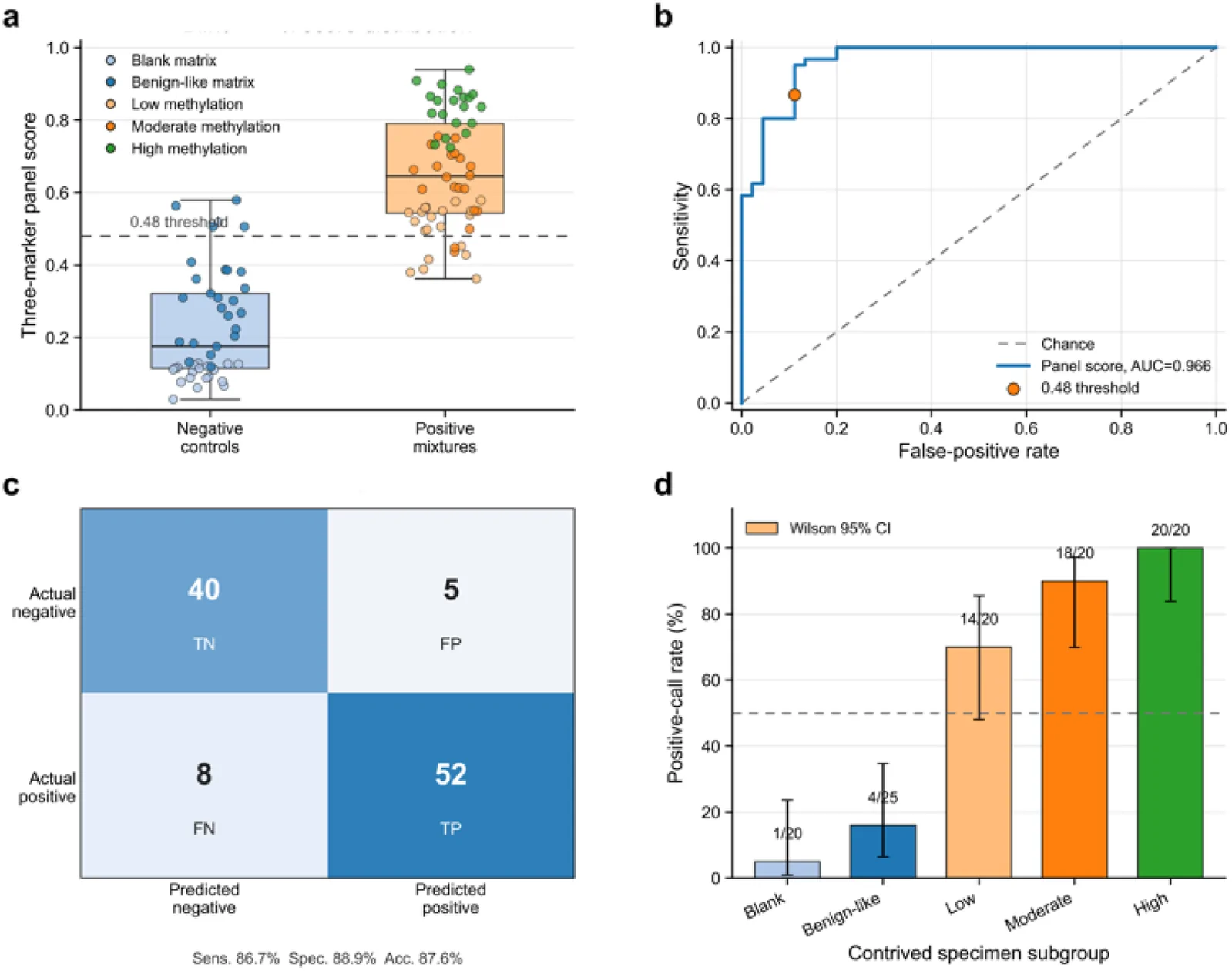
Blind contrived-specimen validation in artificial plasma matrix. (a) Distribution of three-marker methylation scores in methylation-negative artificial-matrix controls and methylation-positive breast-cancer-like specimens. (b) ROC analysis for the combined panel. (c) Confusion matrix at the prespecified 0.48 score threshold. (d) Positive rate by contrived methylation category and matrix-control subgroup.

Blind contrived-specimen validation tested whether analytical gains translated into a classification signal under controlled nonclinical conditions. The combined panel score was higher in methylation-positive breast-cancer-like DNA mixtures than in methylation-negative matrix controls (Fig. 9a). ROC analysis gave an AUC of 0.966 (Fig. 9b). At the fixed 0.48 threshold, the assay identified 52 of 60 methylation-positive specimens and correctly classified 40 of 45 methylation-negative specimens, corresponding to 86.7% positive-call sensitivity, 88.9% specificity, and 87.6% accuracy (Fig. 9c). Positivity increased across low-, moderate-, and high-methylation contrived categories, whereas blank-matrix and benign-like matrix controls showed much lower positivity (Fig. 9d). This pattern validates the analytical classifier without requiring patient recruitment, residual clinical specimens, or institutional ethics approval.

The blind-panel performance should be compared with methylation literature as an analytical model rather than as a clinical diagnostic claim. Mutation assays can be highly specific but may struggle in early-stage disease because a small number of mutant fragments must be sampled from limited plasma DNA.^7,10^ Methylation assays can aggregate information across multiple CpG loci and can exploit tissue-specific epigenetic structure.^17–21^ In Table 2, the present three-gene assay is positioned against cfDNA methylation literature to show how its analytical discrimination relates to reported clinical directions, while making clear that the current validation used contrived specimens. This is the central tradeoff: a targeted electrochemical panel cannot provide the broad tissue localization of sequencing-based methylome tests, but it can provide a fast and ethically low-burden method-development route before any future clinical study is initiated.

**Table 2:** Clinical-context comparison for cfDNA methylation and ctDNA detection

Study	Marker or clinical context	Performance context	Comment
This work	RASSF1A/APC/FOXA1 panel in 60 methylation-positive and 45 methylation-negative contrived specimens	AUC 0.966; positive-call sensitivity 86.7%; specificity 88.9%	—
Salta *et al.*	APC/FOXA1/RASSF1A-based cfDNA methylation in breast cancer and control plasma samples	Reported diagnostic utility with panel-dependent sensitivity and specificity	24
Nunes *et al.*	APC/FOXA1/RASSF1A PanCancer cfDNA methylation panel	72% sensitivity and 74% specificity for major cancers in women	25
Shen *et al.*	Genome-scale plasma cfDNA methylome classification	Large feature space enables sensitive tumour detection and tissue classification	19
Liu *et al.*	Targeted methylation signatures for multi-cancer detection	High specificity and localization potential in cell-free DNA	21
Klein *et al.*	Targeted methylation-based early detection validation	Independent validation of a methylation classifier	17

Table 3 compares the assay with representative NPG, electrochemical CRISPR, and MSRE-Cas12a methods. The platform does not claim the absolute lowest reported LOD, because barcode-amplified NPG systems and optimized fluorescence CRISPR systems can reach very low copy equivalents under controlled conditions 34,45. Its advantage is the combination of methylation selectivity, surface-confined electrochemical readout, fM-level sensitivity, and a three-marker blind-panel score within an approximately 85 min workflow.

**Table 3:** Analytical-performance comparison with representative electrochemical, CRISPR, and NPG nucleic-acid assays

Study	Method and target	Performance	Matrix and refs
This work	NPG-MSRE-RPA-Cas12a-SWV for methylated RASSF1A/APC/FOXA1	LOD 3.4–5.2 fM; linear range 10 fM-10 nM; 85 min workflow	Artificial plasma matrix; this article
Lu *et al.*	MSRE-RPA-Cas12a fluorescence for a methylated DNA model	LOD 0.1 fM; linear range 0.1 fM-10 pM; 65 min workflow	Synthetic DNA; ref. 34
Dong *et al.*	Ratiometric CRISPR/Cas12a electrochemistry for ctDNA	LOD 1.0 pM; linear range 1 pM-10 nM; 120 min workflow	Serum; ref. 52
Zhang *et al.*	Hairpin reporter electrochemical CRISPR assay	LOD 30 pM; linear range 0.1–100 nM; 60 min workflow	Buffer nucleic-acid target; ref. 50
Ge *et al.*	Dual-mode electrochemical CRISPR/Cas12a biosensor	PCR-free assay; electrochemical/lateral mode coupling	Agricultural DNA; ref. 30
Hu *et al.*	NPG electrode with multifunctional DNA-Au bar codes	LOD 28 aM; linear range 0.1 fM-10 pM; 180 min workflow	Buffer DNA target; ref. 45
Zhong *et al.*	NPG-MB DNA biosensor for PML/RARalpha fusion sequence	LOD 6.7 pM; linear range 10 pM-1 nM; 120 min workflow	Clinical RNA-derived DNA; ref. 46

A deeper comparison with prior NPG sensors highlights why the present architecture is distinct. Hu *et al.* 45 achieved attomolar DNA detection by combining NPG with DNA-Au barcode amplification, whereas Zhong *et al.* 46 used MB and NPG for fusion-gene detection with pM-level sensitivity. Those studies established that NPG can amplify nucleic-acid signals, but they did not address methylation discrimination. Daggumati *et al.* 44 showed that NPG can be more resilient than planar gold in biofouling media, which is directly relevant to plasma testing. Matharu *et al.* 48 further demonstrated that NPG morphology alters nucleic-acid biosensor behavior. The present assay builds on these findings by adding an upstream methylation filter and a programmable CRISPR amplification step.

A deeper comparison with CRISPR diagnostics also clarifies the contribution of the electrode. SHERLOCK and DETECTR platforms demonstrated that collateral-cleavage enzymes can support sensitive and portable nucleic-acid detection.^28–33^ However, many CRISPR assays use fluorescence or lateral flow readouts, which are powerful but not always quantitative over a broad dynamic range. Electrochemical CRISPR formats such as hairpin reporter cleavage and ratiometric signal designs provide a route to compact quantification.^50–52^ The NPG interface extends that route by increasing reporter capacity and improving matrix tolerance. In other words, Cas12a supplies molecular specificity after target recognition, while NPG determines how efficiently that molecular event is transduced into a stable current response.

The material data in Fig. 2 should also be interpreted against the competing requirements of clinical biosensors. A high-roughness electrode can increase current, but excessive surface area can increase capacitive background, slow washing, and make electrode-to-electrode normalization more difficult. The 9.4-fold roughness factor observed here sits in a useful middle range and was derived from CV-based ECSA comparison rather than from Nyquist semicircle fitting. It is large enough to improve MB reporter loading relative to planar gold, yet it does not produce an overwhelming nonfaradaic background in SWV. This matters because methylated-DNA measurements rely on percent current loss rather than absolute current alone. If the starting current varies too much between electrodes, the same Cas12a cleavage event could appear as different suppression values. The relatively narrow reproducibility window in Fig. 8 therefore validates the material choice more strongly than a roughness value alone would. In this sense, NPG is not simply a sensitivity enhancer; it is a variance-management material that allows enzymatic signal amplification to be quantified with acceptable precision.

The optimization curves in Fig. 4 further reveal why the method should be tuned as an integrated assay rather than as independent modules. Increasing HpaII digestion time improves background removal only until digestion is complete; beyond that point it can lengthen workflow without improving discrimination. Extending RPA improves target yield but can eventually increase nonspecific products, which are particularly problematic for CRISPR readouts because collateral cleavage converts rare activation events into amplified reporter loss. Increasing Cas12a concentration raises cleavage rate but also increases reagent cost and can expose the surface to higher nonspecific nuclease background. Increasing reporter density raises baseline current but can reduce cleavage accessibility. These linked tradeoffs explain why the selected conditions are not the maximum value in every panel. The final operating point prioritizes the full diagnostic objective: a stable threshold that preserves specificity in plasma while maintaining fM-level detection.

The RASSF1A data in Fig. 5 also illustrate the difference between analytical sensitivity and clinically meaningful sensitivity. A 3.4 fM LOD demonstrates that the electrochemical CRISPR circuit can detect very low target abundance, but early breast cancer detection depends on whether methylated fragments are consistently present and biologically specific. RASSF1A is attractive because promoter methylation has been reported across breast cancer cohorts and meta-analyses.^22,23^ Still, RASSF1A alone cannot represent the molecular heterogeneity of breast cancer. The allele-fraction experiment therefore matters as much as the calibration curve. It shows that a low methylated fraction can be distinguished when total DNA is dominated by unmethylated molecules, a condition closer to plasma than purified target standards. This is why the analytical section cannot be reduced to LOD ranking. The more meaningful claim is that low LOD, low cross-reactivity, and allele-fraction discrimination occur together under one assay architecture.

The panel results in Fig. 6 and Table 1 should be viewed through the lens of breast cancer heterogeneity. Breast cancer includes hormone receptor-positive, HER2-positive, and triple-negative disease, each with distinct biology and tumor shedding patterns.^4^ A single epigenetic marker may perform well in one subtype but poorly in another. The use of RASSF1A, APC, and FOXA1 creates a compact panel that samples tumor-suppressor silencing, Wnt-pathway-associated methylation, and lineage-related transcriptional regulation. This does not make the panel exhaustive, but it improves resilience against marker dropout. The low cross-reactivity in Fig. 6c is therefore important because it preserves marker independence. If APC activation were caused by RASSF1A target or if FOXA1 crRNA responded broadly to non-target fragments, a three-marker score would give a false impression of biological breadth. The observed orthogonality allows the panel score to reflect combined methylation evidence rather than technical crosstalk.

The specificity and recovery experiments in Fig. 7 add another layer of comparison with sequencing-based methylation assays. Sequencing assays can computationally correct for many sources of background, but they require library preparation, sufficient molecule counts, and bioinformatic normalization.^17,19–21^ An electrochemical assay has fewer computational degrees of freedom; it must reject interference physically and enzymatically before the signal reaches the electrode. The low response to unmethylated DNA shows that the HpaII gate performs the first rejection step. The low response to single-base mismatch and non-target cfDNA-like fragments shows that crRNA recognition performs the second. The preserved signal in albumin, ascorbate, and artificial plasma matrix shows that the electrode performs the third. This layered specificity is a key mechanistic advantage. It distributes error control across chemistry, enzymology, and materials engineering instead of placing the entire burden on one molecular recognition event.

The blind-panel results in Fig. 9 should not be interpreted only by the AUC value. AUC summarizes ranking performance across thresholds, but practical assay development also requires a prespecified threshold that balances false positives against false negatives. At the fixed 0.48 panel threshold, the assay produced five false positives and eight false negatives in contrived specimens. These errors are useful because they show where analytical ambiguity emerges: samples near the threshold are most sensitive to small changes in methylated fraction, reporter density, and digestion efficiency. This error structure suggests how the assay should be advanced. It should first be strengthened with additional nonclinical controls and blinded contrived panels, then moved to authentic clinical materials only after an ethics-approved protocol and sample-governance plan are in place.

The literature comparison in Table 3 emphasizes that no single metric determines assay quality. The barcode-amplified NPG approach reported by Hu *et al.*^45^ provides extraordinary analytical sensitivity but requires a more elaborate amplification architecture. Fluorescence MSRE-RPA-Cas12a can reach very low LODs and is elegant for methylation detection, but electrochemical readout may be easier to miniaturize for low-cost readers.^34^ Ratiometric CRISPR electrochemistry improves reliability by comparing two signals, but it can require more complex probe design and signal processing.^52^ The present assay occupies a middle position: it is not the lowest-LOD system in the table, but it integrates methylation selectivity, a compact electrode readout, artificial-matrix recovery, and an interpretable three-marker score. For translational biosensing, that balanced profile may be more useful than optimizing a single benchmark under ideal buffer conditions.

The assay also raises practical manufacturing questions. NPG morphology is sensitive to alloy composition, dealloying time, temperature, and post-treatment, and each factor can shift pore diameter and ligament connectivity.^40–43^ For a deployable test, these parameters would need lot-release criteria such as roughness factor, MB reporter loading, blank current range, and control-target suppression. The reproducibility data in Fig. 8 suggest that such criteria are achievable, but they also show where quality control should focus. Batch-to-batch shifts of only a few percentage points could influence specimens near the analytical threshold. A future cartridge should therefore include an internal process control, a methylated positive control, and a digestion control for unmethylated DNA. These controls would make threshold decisions more robust and would help distinguish borderline contrived specimens from technical drift.

Preanalytical control is deliberately outside the present experimental scope because it would require human-derived samples and ethics review. Nevertheless, the assay was designed with those later constraints in mind. Blood-collection tube chemistry, time to plasma separation, centrifugation protocol, storage temperature, freeze-thaw history, and extraction method can influence total cfDNA yield and leukocyte DNA contamination.^5,6,8,9^ For methylation assays, leukocyte contamination is especially relevant because it can dilute tumor-derived fragments and introduce background methylation patterns unrelated to breast tumors. The current artificial-matrix workflow cannot resolve those clinical variables, but it can establish analytical thresholds, interference tolerance, and reporter stability before ethically governed sample access is sought. A useful next nonclinical study would vary artificial matrix composition, synthetic background DNA load, and fragment-length distribution to stress-test the 0.48 threshold without using patient material.

The assay's quantitative format also creates opportunities for future longitudinal study, but those applications should be separated from the present ethics-free method development. A binary positive or negative result may be sufficient for a contrived panel, whereas a continuous panel score could later track changes over time if approved clinical samples become available. In a future ethically reviewed study, repeated low-level increases across RASSF1A, APC, and FOXA1 might be more informative than a single borderline measurement. In treated patients, declining methylation score could complement imaging and conventional biomarkers, while renewed elevation might suggest molecular recurrence before radiographic change. That potential parallels the use of ctDNA for monitoring metastatic breast cancer,^5^ but it cannot be claimed from the current nonclinical dataset. The current contribution is to establish a stable quantitative platform that could support such studies once sample governance is in place.

Finally, the method has implications beyond breast cancer if the marker set is redesigned. HpaII-compatible methylation sites occur in many promoter and enhancer regions, and Cas12a crRNAs can be reprogrammed for other protected amplification products. The electrode and reporter chemistry could therefore remain constant while the biological panel changes. This modularity is valuable because methylation biomarkers are disease- and tissue-specific. In prostate, colorectal, lung, or gynecologic cancers, the same NPG-MSRE-RPA-Cas12a framework could be adapted to other promoter panels, provided that digestion sites, amplification primers, and crRNAs are redesigned and clinically validated. The present breast cancer panel thus serves as a demonstration of an assay architecture rather than a closed marker system. Its most transferable feature is the division of labor: methylation enzymes select epigenetic state, RPA and Cas12a amplify target recognition, and NPG translates nuclease activity into a portable electrochemical signal.

From a future clinical-study perspective, the most informative validation design would not only compare cancer and healthy samples, but that step should occur only after ethics approval. It should include benign breast lesions, inflammatory disease, dense-breast imaging recalls, age-matched controls, and longitudinal follow-up for initially negative participants. Those groups would test whether the panel discriminates malignant methylation from clinically common confounders. Before that stage, the assay should be benchmarked against an orthogonal nonclinical methylation method, such as methylation-specific PCR or targeted bisulfite sequencing of synthetic standards, so that electrode signal can be traced back to molecular methylation status. This comparison would help determine whether discordant contrived specimens arise from enzymatic digestion, amplification bias, or electrochemical transduction.

The main limitation is that the assay remains targeted and has not been evaluated with human specimens. A three-marker panel can miss tumors that do not methylate the selected promoters and can produce positives in benign conditions if methylation background rises with age, inflammation, or non-breast pathology. Large methylome classifiers address this limitation with thousands of features, but they require sequencing infrastructure and more complex validation.^17,19–21^ A rational path forward is therefore tiered rather than competitive: compact electrochemical panels can be optimized first with synthetic and artificial-matrix models, while sequencing-based methylome tests and authentic clinical specimens can be reserved for later ethically approved validation. Future work should evaluate leukocyte methylation background, extraction yield, hemolysis, and longitudinal stability only under an approved human-subjects protocol.

## 4. Conclusion

This study presents a NPG electrode-assisted CRISPR/Cas12a assay for synthetic methylated DNA models that represent breast-cancer-associated promoter fragments. NPG increased ECSA by 9.4-fold and supported dense MB-reporter immobilization. HpaII digestion provided methylation selectivity, RPA supplied low-temperature amplification, and Cas12a converted protected methylated targets into catalytic reporter cleavage. The resulting assay achieved fM-level LODs across RASSF1A, APC, and FOXA1, resolved 1% methylated DNA, and maintained acceptable recovery, precision, and short-term storage stability in artificial-matrix testing.

The blind contrived-specimen panel reached an AUC of 0.966 with balanced positive-call sensitivity and specificity in artificial plasma matrix. Mechanistically, the performance reflects the coupling of a porous antifouling electrode, an enzymatic methylation gate, programmable CRISPR amplification, and a quantitative SWV readout. The most important next steps are additional nonclinical robustness testing, orthogonal methylation confirmation using synthetic standards, and only then ethically approved validation with authentic extracted cfDNA. If those later studies confirm the observed trends, the platform could contribute to accessible methylated-cfDNA assay development as an adjunct to imaging-based early breast cancer screening.

## Author contributions

X. W. conceived the study and designed the experiments; F. Z. and H. Y. developed the methodology and performed the experimental investigation; J. W. and X. L. conducted data curation and formal analysis; F. K. contributed to validation and visualization; X. W. drafted the original manuscript; Y. Z. supervised the project, contributed to writing – review & editing, and provided project administration and funding acquisition. All authors have read and agreed to the published version of the manuscript.

## Conflicts of interest

There are no conflicts to declare.

## Data Availability

Data are available upon request from the authors.

## References

[cit1] Sung H., Ferlay J., Siegel R. L., Laversanne M., Soerjomataram I., Jemal A., Bray F. (2021). Ca-Cancer J. Clin..

[cit2] Bray F., Laversanne M., Sung H., Ferlay J., Siegel R. L., Soerjomataram I., Jemal A. (2024). Ca-Cancer J. Clin..

[cit3] Giaquinto A. N., Sung H., Newman L. A., Freedman R. A., Smith R. A., Star J., Jemal A., Siegel R. L. (2024). Ca-Cancer J. Clin..

[cit4] Harbeck N., Penault-Llorca F., Cortes J., Gnant M., Houssami N., Poortmans P., Ruddy K., Tsang J., Cardoso F. (2019). Nat. Rev. Dis. Primers.

[cit5] Bettegowda C., Sausen M., Leary R. J., Kinde I., Wang Y., Agrawal N., Bartlett B. R., Wang H., Luber B., Alani R. M., Antonarakis E. S., Azad N. S., Bardelli A., Brem H., Cameron J. L., Lee C. C., Fecher L. A., Gallia G. L., Gibbs P., Le D., Giuntoli R. L., Goggins M., Hogarty M. D., Holdhoff M., Hong S.-M., Jiao Y., Juhl H. H., Kim J. J., Siravegna G., Laheru D. A., Lauricella C., Lim M., Lipson E. J., Marie S. K. N., Netto G. J., Oliner K. S., Olivi A., Olsson L., Riggins G. J., Sartore-Bianchi A., Schmidt K., Shih le-M., Oba-Shinjo S. M., Siena S., Theodorescu D., Tie J., Harkins T. T., Veronese S., Wang T.-L., Weingart J. D., Wolfgang C. L., Wood L. D., Xing D., Hruban R. H., Wu J., Allen P. J., Schmidt C. M., Choti M. A., Velculescu V. E., Kinzler K. W., Vogelstein B., Papadopoulos N., Diaz L. A. (2014). Sci. Transl. Med..

[cit6] Dawson S.-J., Tsui D. W. Y., Murtaza M., Biggs H., Rueda O. M., Chin S.-F., Dunning M. J., Gale D., Forshew T., Mahler-Araujo B., Rajan S., Humphray S., Becq J., Halsall D., Wallis M., Bentley D., Caldas C., Rosenfeld N. (2013). N. Engl. J. Med..

[cit7] Heitzer E., Haque I. S., Roberts C. E. S., Speicher M. R. (2019). Nat. Rev. Genet..

[cit8] Wan J. C. M., Massie C., Garcia-Corbacho J., Mouliere F., Brenton J. D., Caldas C., Pacey S., Baird R., Rosenfeld N. (2017). Nat. Rev. Cancer.

[cit9] Schwarzenbach H., Hoon D. S. B., Pantel K. (2011). Nat. Rev. Cancer.

[cit10] Li M., Wang C., Yu B., Zhang X., Shi F., Liu X. (2019). Biosci. Rep..

[cit11] Jones P. A. (2012). Nat. Rev. Genet..

[cit12] Laird P. W. (2010). Nat. Rev. Genet..

[cit13] Jones P. A., Issa J.-P. J., Baylin S. (2016). Nat. Rev. Genet..

[cit14] Esteller M. (2008). N. Engl. J. Med..

[cit15] Hanahan D. (2022). Cancer Discovery.

[cit16] Baylin S. B., Jones P. A. (2016). Cold Spring Harbor Perspect. Biol..

[cit17] Klein E. A., Richards D., Cohn A., Tummala M., Lapham R., Cosgrove D., Chung G., Clement J., Gao J., Hunkapiller N., Jamshidi A., Kurtzman K. N., Seiden M. V., Swanton C., Liu M. C. (2021). Ann. Oncol..

[cit18] Cristiano S., Leal A., Phallen J., Fiksel J., Adleff V., Bruhm D. C., Jensen S. Ø., Medina J. E., Hruban C., White J. R., Palsgrove D. N., Niknafs N., Anagnostou V., Forde P., Naidoo J., Marrone K., Brahmer J., Woodward B. D., Husain H., Van Rooijen K. L., Ørntoft M.-B. W., Madsen A. H., Van De Velde C. J. H., Verheij M., Cats A., Punt C. J. A., Vink G. R., Van Grieken N. C. T., Koopman M., Fijneman R. J. A., Johansen J. S., Nielsen H. J., Meijer G. A., Andersen C. L., Scharpf R. B., Velculescu V. E. (2019). Nature.

[cit19] Shen S. Y., Singhania R., Fehringer G., Chakravarthy A., Roehrl M. H. A., Chadwick D., Zuzarte P. C., Borgida A., Wang T. T., Li T., Kis O., Zhao Z., Spreafico A., Medina T. D. S., Wang Y., Roulois D., Ettayebi I., Chen Z., Chow S., Murphy T., Arruda A., O'Kane G. M., Liu J., Mansour M., McPherson J. D., O'Brien C., Leighl N., Bedard P. L., Fleshner N., Liu G., Minden M. D., Gallinger S., Goldenberg A., Pugh T. J., Hoffman M. M., Bratman S. V., Hung R. J., De Carvalho D. D. (2018). Nature.

[cit20] Moss J., Magenheim J., Neiman D., Zemmour H., Loyfer N., Korach A., Samet Y., Maoz M., Druid H., Arner P., Fu K.-Y., Kiss E., Spalding K. L., Landesberg G., Zick A., Grinshpun A., Shapiro A. M. J., Grompe M., Wittenberg A. D., Glaser B., Shemer R., Kaplan T., Dor Y. (2018). Nat. Commun..

[cit21] Liu M. C., Oxnard G. R., Klein E. A., Swanton C., Seiden M. V., Liu M. C., Oxnard G.
R., Klein E. A., Smith D., Richards D., Yeatman T. J., Cohn A. L., Lapham R., Clement J., Parker A. S., Tummala M. K., McIntyre K., Sekeres M. A., Bryce A. H., Siegel R., Wang X., Cosgrove D. P., Abu-Rustum N. R., Trent J., Thiel D. D., Becerra C., Agrawal M., Garbo L. E., Giguere J. K., Michels R. M., Harris R. P., Richey S. L., McCarthy T. A., Waterhouse D. M., Couch F. J., Wilks S. T., Krie A. K., Balaraman R., Restrepo A., Meshad M. W., Rieger-Christ K., Sullivan T., Lee C. M., Greenwald D. R., Oh W., Tsao C.-K., Fleshner N., Kennecke H. F., Khalil M. F., Spigel D. R., Manhas A. P., Ulrich B. K., Kovoor P. A., Stokoe C., Courtright J. G., Yimer H. A., Larson T. G., Swanton C., Seiden M. V., Cummings S. R., Absalan F., Alexander G., Allen B., Amini H., Aravanis A. M., Bagaria S., Bazargan L., Beausang J. F., Berman J., Betts C., Blocker A., Bredno J., Calef R., Cann G., Carter J., Chang C., Chawla H., Chen X., Chien T. C., Civello D., Davydov K., Demas V., Desai M., Dong Z., Fayzullina S., Fields A. P., Filippova D., Freese P., Fung E. T., Gnerre S., Gross S., Halks-Miller M., Hall M. P., Hartman A.-R., Hou C., Hubbell E., Hunkapiller N., Jagadeesh K., Jamshidi A., Jiang R., Jung B., Kim T., Klausner R. D., Kurtzman K. N., Lee M., Lin W., Lipson J., Liu H., Liu Q., Lopatin M., Maddala T., Maher M. C., Melton C., Mich A., Nautiyal S., Newman J., Newman J., Nicula V., Nicolaou C., Nikolic O., Pan W., Patel S., Prins S. A., Rava R., Ronaghi N., Sakarya O., Satya R. V., Schellenberger J., Scott E., Sehnert A. J., Shaknovich R., Shanmugam A., Shashidhar K. C., Shen L., Shenoy A., Shojaee S., Singh P., Steffen K. K., Tang S., Toung J. M., Valouev A., Venn O., Williams R. T., Wu T., Xu H. H., Yakym C., Yang X., Yecies J., Yip A. S., Youngren J., Yue J., Zhang J., Zhang L., (Quan) Zhang L., Zhang N., Curtis C., Berry D. A. (2020). Ann. Oncol..

[cit22] Jiang Y., Cui L., Chen W., Shen S., Ding L. (2012). PLoS One.

[cit23] Gootenberg J. S., Abudayyeh O. O., Lee J. W., Essletzbichler P., Dy A. J., Joung J., Verdine V., Donghia N., Daringer N. M., Freije C. A., Myhrvold C., Bhattacharyya R. P., Livny J., Regev A., Koonin E. V., Hung D. T., Sabeti P. C., Collins J. J., Zhang F. (2017). Science.

[cit24] Salta S., Nunes S. P., Fontes-Sousa M., Lopes P., Freitas M., Caldas M., Antunes L., Castro F., Antunes P., Palma De Sousa S., Henrique R., Jerónimo C. (2018). JCM.

[cit25] Nunes S., Moreira-Barbosa C., Salta S., Palma De Sousa S., Pousa I., Oliveira J., Soares M., Rego L., Dias T., Rodrigues J., Antunes L., Henrique R., Jerónimo C. (2018). Cancers.

[cit26] Liu Y., Siejka-Zielińska P., Velikova G., Bi Y., Yuan F., Tomkova M., Bai C., Chen L., Schuster-Böckler B., Song C.-X. (2019). Nat. Biotechnol..

[cit27] Herman J. G., Graff J. R., Myöhänen S., Nelkin B. D., Baylin S. B. (1996). Proc. Natl. Acad. Sci. U. S. A..

[cit28] Chen J. S., Ma E., Harrington L. B., Da Costa M., Tian X., Palefsky J. M., Doudna J. A. (2018). Science.

[cit29] Li S.-Y., Cheng Q.-X., Wang J.-M., Li X.-Y., Zhang Z.-L., Gao S., Cao R.-B., Zhao G.-P., Wang J. (2018). Cell Discovery.

[cit30] Broughton J. P., Deng X., Yu G., Fasching C. L., Servellita V., Singh J., Miao X., Streithorst J. A., Granados A., Sotomayor-Gonzalez A., Zorn K., Gopez A., Hsu E., Gu W., Miller S., Pan C.-Y., Guevara H., Wadford D. A., Chen J. S., Chiu C. Y. (2020). Nat. Biotechnol..

[cit31] Ge H., Wang X., Xu J., Lin H., Zhou H., Hao T., Wu Y., Guo Z. (2021). Anal. Chem..

[cit32] Gootenberg J. S., Abudayyeh O. O., Kellner M. J., Joung J., Collins J. J., Zhang F. (2018). Science.

[cit33] Kellner M. J., Koob J. G., Gootenberg J. S., Abudayyeh O. O., Zhang F. (2019). Nat. Protoc..

[cit34] Lu Z., Ye Z., Li P., Jiang Y., Han S., Ma L. (2024). Biosensors.

[cit35] Liu W.-J., Wang L.-Y., Ma F., Zhang C.-Y. (2025). Adv. Sci..

[cit36] Chen L.-Y., Zhang Q., Liu W.-J., Zhang C.-Y. (2026). Sens. Actuators, B.

[cit37] Guo J., Zhao M., Chen C., Wang F., Chen Z. (2024). Analyst.

[cit38] Bao J., Ding K., Zhu Y. (2023). Anal. Biochem..

[cit39] Chen Y., Wang X., Luo S., Dai C., Wu Y., Zhao J., Liu W., Kong D., Yang Y., Geng L., Liu Y., Wei D. (2024). Anal. Chem..

[cit40] Ahangar L. E., Mehrgardi M. A. (2012). Biosens. Bioelectron..

[cit41] Seker E., Reed M. L., Begley M. R. (2009). Materials.

[cit42] Scanlon M. D., Salaj-Kosla U., Belochapkine S., MacAodha D., Leech D., Ding Y., Magner E. (2012). Langmuir.

[cit43] Lang X.-Y., Fu H.-Y., Hou C., Han G.-F., Yang P., Liu Y.-B., Jiang Q. (2013). Nat. Commun..

[cit44] Daggumati P., Matharu Z., Wang L., Seker E. (2015). Anal. Chem..

[cit45] Hu K., Lan D., Li X., Zhang S. (2008). Anal. Chem..

[cit46] Zhong G., Liu A., Chen X., Wang K., Lian Z., Liu Q., Chen Y., Du M., Lin X. (2011). Biosens. Bioelectron..

[cit47] Patel J., Radhakrishnan L., Zhao B., Uppalapati B., Daniels R. C., Ward K. R., Collinson M. M. (2013). Anal. Chem..

[cit48] Matharu Z., Daggumati P., Wang L., Dorofeeva T. S., Li Z., Seker E. (2017). ACS Appl. Mater. Interfaces.

[cit49] Piepenburg O., Williams C. H., Stemple D. L., Armes N. A. (2006). PLoS Biol..

[cit50] Zhang D., Yan Y., Que H., Yang T., Cheng X., Ding S., Zhang X., Cheng W. (2020). ACS Sens..

[cit51] Liu J., Wan Q., Zeng R., Tang D. (2021). Anal. *Methods*.

[cit52] Dong J., Li X., Hou C., Hou J., Huo D. (2024). Anal. Chem..

[cit53] Downs A. M., Gerson J., Hossain M. N., Ploense K., Pham M., Kraatz H.-B., Kippin T., Plaxco K. W. (2021). ACS Sens..

[cit54] Drummond T. G., Hill M. G., Barton J. K. (2003). Nat. Biotechnol..

[cit55] Fan C., Plaxco K. W., Heeger A. J. (2003). Proc. Natl. Acad. Sci. U. S. A..

